# Transcriptome-associated metabolomics reveals the molecular mechanism of flavonoid biosynthesis in *Desmodium styracifolium* (Osbeck.) Merr under abiotic stress

**DOI:** 10.3389/fpls.2024.1431148

**Published:** 2024-08-19

**Authors:** Hongyang Gao, Xi Huang, Pengfei Lin, Yuqing Hu, Ziqi Zheng, Quan Yang

**Affiliations:** ^1^ School of Chinese Materia Medica, Guangdong Pharmaceutical University, Guangzhou, China; ^2^ Shenzhen Traditional Chinese Medicine Manufacturing Innovation Ceter Co., Ltd., Shenzhen, China; ^3^ Guangdong Provincial Research Center on Good Agricultural Practice & Comprehensive Agricultural Development Engineering Technology of Cantonese Medicinal Materials, Guangzhou, China; ^4^ Comprehensive Experimental Station of Guangzhou, Chinese Material Medica, China Agriculture Research System (CARS-21-16), Guangzhou, China; ^5^ Key Laboratory of State Administration of Traditional Chinese Medicine for Production & Development of Cantonese Medicinal Materials, Guangzhou, China

**Keywords:** *Desmodium styracifolium* (Osbeck.) Merr., flavonoids, transcriptomics, metabolomics, transcription factor

## Abstract

The primary pharmacological components of *Desmodium styracifolium* (Osbeck.) Merr. are flavonoids, which have a broad range of pharmacological effects and are important in many applications. However, there have been few reports on the molecular mechanisms underlying flavonoid biosynthesis in the pharmacodynamic constituents of *D. styracifolium*. Flavonoid biosynthesis in *D. styracifolium* pharmacodynamic constituents has, however, been rarely studied. In this study, we investigated how salt stress, 6-BA (6-Benzylaminopurine) treatment, and PEG 6000-simulated drought stress affect flavonoid accumulation in *D. styracifolium* leaves. We integrated metabolomics and transcriptomic analysis to map the secondary metabolism regulatory network of *D. styracifolium* and identify key transcription factors involved in flavonoid biosynthesis. We then constructed overexpression vectors for the transcription factors and used them to transiently infiltrate *Nicotiana benthamiana* for functional validation. This experiment confirmed that the transcription factor DsMYB60 promotes the production of total flavonoids in *Nicotiana tabacum* L. leaves. This study lays the foundation for studying flavonoid biosynthesis in *D. styracifolium* at the molecular level. Furthermore, this study contributes novel insights into the molecular mechanisms involved in the biosynthesis of active ingredients in medicinal plants.

## 1 Introduction

The genus *Desmodium styracifolium (Osbeck.) Merr.*, belonging to the Leguminosae family, is part of the Locust family. It has been used by practitioners of traditional Chinese medicine to treat many different diseases, and in 1977, it was finally added to the Chinese Pharmacopoeia under the name “Guangjinqincao” (Yen et al., 2023). *Desmodium styracifolium* has many beneficial medicinal properties, including antioxidant (Li et al., 2022c) and anti-kidney stone (Hou et al., 2018) effects, as well as preventive effects against rheumatoid arthritis (Noh et al., 2017). Furthermore, it also exhibits anti-pathogenic effects, allowing it to resist plant pathogens (Quang et al., 2022). *Desmodium styracifolium* is currently used across many industries, including the food and medical industries, and it has gained increasing recognition for its potential value in clinical applications; thus, *D. styracifolium* has a high economic, medicinal, and biological value.

Researchers have found *D. styracifolium* contains a variety of chemical constituents, including flavonoids, alkaloids, phenols, tannins, polysaccharides, and volatile oils (Acharya et al., 2023). Of these, flavonoids are the primary bioactive components of *D. styracifolium*, and their content in the plant has a direct effect on their efficacy. Currently, the main flavonoids that are found in *D. styracifolium* extracts include schaftoside, isovitexin, isoschaftoside, and isoorientin (Li et al., 2022a). The schaftoside content, in particular, can be used as an index to reflect the quality of *D. styracifolium*. However, the molecular mechanisms of this plant’s flavonoid biosynthesis have yet to be fully elucidated.

Second-generation sequencing methods, which have been increasingly well developed in recent years, have made the analysis of large amounts of transcriptomic and metabolomic data possible. As a result, key genes involved in primary and secondary metabolism have been identified (Li et al., 2023; Shu et al., 2023; Yang et al., 2023). Metabolite synthesis and catabolism are coordinated by a number of enzymes and their associated genes. Therefore, changes in metabolite content may result in corresponding trends in the expression of these genes (Omranian et al., 2015; Zhang M. et al., 2023). Therefore, joint metabolomic and transcriptomic analyses can be used to detect correlations between the expression of unknown genes and metabolite levels, as well as co-expression with known functional enzyme genes. According to correlation analyses, a previously unknown gene may contribute to substance metabolism (Li et al., 2020; Yin et al., 2022). For example, Yuan et al. (Yuan et al., 2022) used a combination of transcriptomics and metabolomics analyses to find that key genes for flavonol synthesis were highly expressed in the stems of a two-year sample of *Dendrobium officinale* Kimura et Migo (Orchidaceae), a higher level of flavonols is accumulated as a result. Similarly, Song et al. (Song et al., 2023) used a combination of metabolomic and transcriptomic analyses to explore the metabolic profiles of flavonoids and their related gene regulatory networks in different tissues of *Hippophae rhamnoides* L. (Elaeagnaceae). They found that the overexpression of *HrMYB114* in transgenic hairy roots significantly increased the flavonoid content and expression of structural genes in the flavonoid synthesis pathway. Li et al. (Li et al., 2024) identified three characteristic flavonoids of *Glycyrrhiza glabra* using integrated metabolic and transcriptomic analyses, and they found that *GibHLH69*, a key transcription factor in the regulatory network, promotes the accumulation of flavonoids, such as *Glycyrrhiza chalcone* A, in *Glycyrrhiza inflata* Batal. by facilitating the expression of *GiCHS20*. While regulatory genes for flavonoid synthesis have been identified in medicinal plants such as *Areca catechu* L. (Palmae) (Lai et al., 2023), *Malus spectabilis* (Ait.) Borkh (Han et al., 2020), and *Ginkgo biloba* L (Liu et al., 2023), no studies have reported the identification of these genes in *D. styracifolium*.

Secondary metabolites, such as glycyrrhetinic acid and flavonoids, have been demonstrated to play important roles in plant responses to abiotic stresses (Yao et al., 2022). 6-BA is a cytokinin that plays an significant role in the synthesis and accumulation of secondary metabolites (Ren et al., 2018). The Integration of different histologies is useful for exploring abiotic stress responses in plants (Ma et al., 2020; Miao et al., 2021).

Therefore, in this study, we focused on developing a secondary metabolic regulatory network for *D. styracifolium* by using joint metabolomics and transcriptomics analysis. Moreover, the key transcription factors involved in flavonoid biosynthesis were also identified, and overexpression vectors were constructed, and performed transient infestation of *Nicotiana benthamiana* to functionally validate the transcription factors. These results indicated that the transcription factor called DsMYB60 significantly affected tobacco leaf flavonoid synthesis. This will lay the foundation for further research into the molecular mechanisms of flavonoid biosynthesis in *D. styracifolium*.

## 2 Results

### 2.1 Effects of 6-BA, PEG 6000, and NaCl on the total flavonoid and flavonoid glycoside contents of *D. styracifolium*

**Figure 1:**
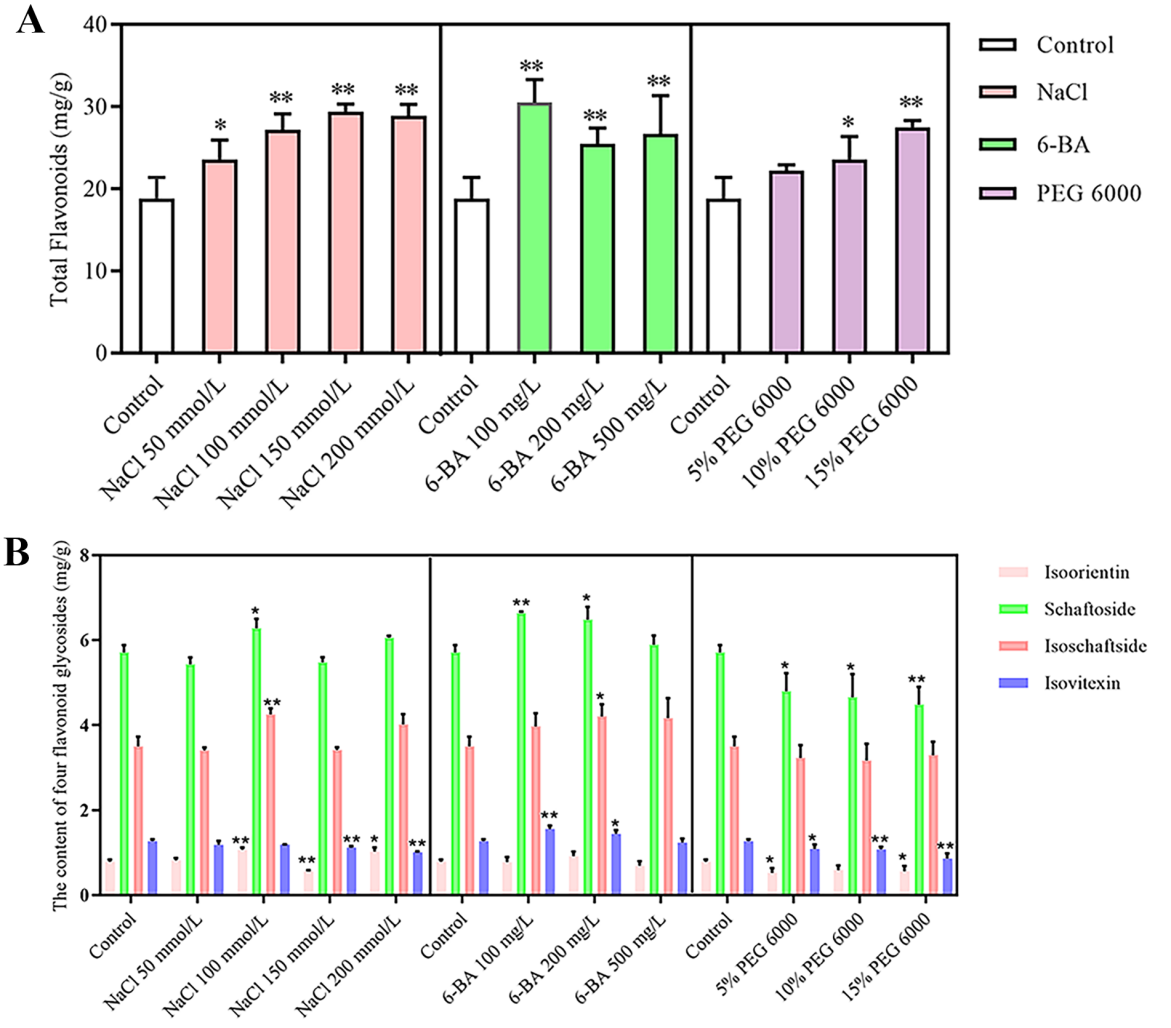
Content of total flavonoids and four flavonoid glycosides of *Desmodium styracifolium* (Osbeck) Merr under different treatments (n = 3) **(A)** Content of total flavonoids of Desmodium styracifolium (Osbeck) Merr under different treatments. **(B)** Content of four flavonoid glycosides of Desmodium styracifolium (Osbeck) Merr under different treatments. (Note: Compared to the control group, *P < 0.05; **P < 0.01, the results were statistically and analytically analyzed using SPSS 21.0 software and plotted using Graphpad Prism 5).

The total flavonoid and schaftoside, isovitexin, isoschaftoside, and isoorientin contents were determined by treating *D. styracifolium* with different concentrations of PEG 6000, 6-BA, and NaCl up to the harvesting stage. The results, which are shown in **Figure 1**, indicate that the total flavonoid content in the leaves of the plants increased significantly after stress, except for in the 5% PEG 6000 group. Among those treated with 6-BA at 100 mg/L, the greatest content was observed, reaching a value of 30.48 mg/g. Moreover, 6-BA treatment promoted the accumulation of schaftoside, isovitexin and isoschaftoside; it increased the contents of schaftoside and isovitexin by 1.16-fold and 1.22-fold, respectively, while the content of isoschaftoside increased by 1.2-fold using 100 mg/L 6-BA as a treatment. The accumulation of isoorientin was significantly inhibited by 5% PEG 6000 stress, resulting in a 33.54% decrease in its content. Additionally, 15% PEG 6000 stress significantly inhibited the accumulation of schaftoside and isovitexin, resulting in 21.45% and 31.96% decreases in their contents, respectively. Salt stress primarily affected the accumulation of isovitexin and isoorientin in *D. styracifolium*.

### 2.2 Data analysis of the metabolome of *D. styracifolium* after 6-BA treatment and PEG 6000 stress

The composition of metabolites in the leaves of *D. styracifolium* was analyzed using non-targeted metabolomics in three groups: the 100 mg/L 6-BA-treated group, the 15% PEG 6000-stressed group, and the control group. Comparatively to the control group, we identified 238 and 4430 differential metabolites in the groups treated with 100 mg/L 6-BA and 15% stressed with PEG 6000, respectively. The number of metabolites in these two groups with upregulated/downregulated expressions were 153/85 and 2100/2330, respectively.

**Figure 2:**
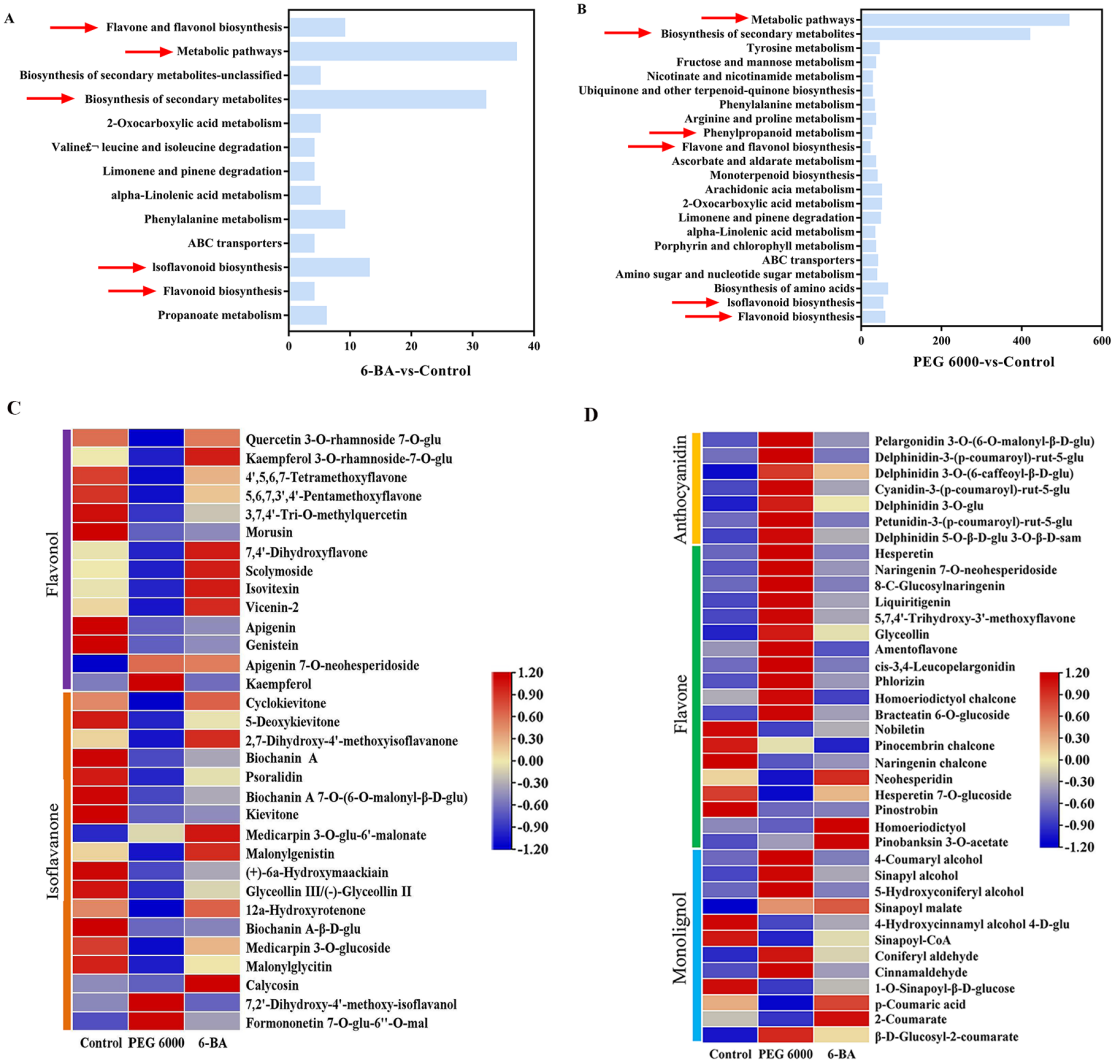
Analysis of differential metabolites. **(A)** KEGG enrichment analysis of the differential metabolites between 6-BA and the control.(The horizontal coordinates represent the number of differential metabolites). **(B)** KEGG enrichment analysis of the differential metabolites between PEG 6000 and the control. (The horizontal coordinates represent the number of differential metabolites). **(C, D)** Differential flavonoid metabolites under 6-BA and PEG 6000 stress (Note: Each column represents a group of samples, each row represents a metabolite, and the metabolite content is indicated by the metabolite peak area z-score followed by the metabolite content; red indicates that the metabolite is highly expressed in the samples, and blue indicates that the metabolite is less highly expressed).

KEGG pathway enrichment analysis was performed on the different metabolites; the results are shown in **Figures 2A, B**. Differential metabolites were primarily enriched in flavonoid, flavonol, and isoflavone biosynthesis, as well as phenylpropane metabolism. Subsequently, we removed isomers from substances enriched in the flavonoid pathway so that the compounds had unique ion IDs and KEGG database numbers, and obtained 70 flavonoid compounds with more significant differences, after which a heatmap was visualized and used for intergroup comparisons (**Figures 2C, D**). A significant increase in flavonol content(Apigenin 7-O-neohesperidoside, Kaempferol 3-O-rhsmnoside-7-O-glucoside,Isovitexin and Vicenin-2, etc.) was observed after treatment with 6-BA in this study. After exposure to PEG 6000 stress, anthocyanins and flavonoids increased significantly, while flavonols and isoflavonoids decreased.

### 2.3 Analysis of data from the transcriptome of *D. styracifolium* after 6-BA treatment and PEG 6000 stress

The sequencing data were analyzed and assessed for quality. A total of 57.52 GB of data were obtained, with Q20 ranging from 96.98% to 97.41% and Q30 > 92.15% (**Supplementary Table S1**). After filtering the data, 61,871 unigenes were identified. The total length, average length, N50 length, and GC content were 88,615,418 bp, 1432 bp, 2088 bp, and 40.30%, respectively (**Supplementary Figure S1**). Sequencing data of high quality were indicated from these results, allowing subsequent analyses to be carried out. Our validation of the RNA-seq data involved screening 16 differentially expressed genes based on the transcriptome data and detecting their expression using the quantitative PCR method. The results of the qPCR validation indicated that the transcriptome data were reliable (**Supplementary Figure S2**).

**Figure 3:**
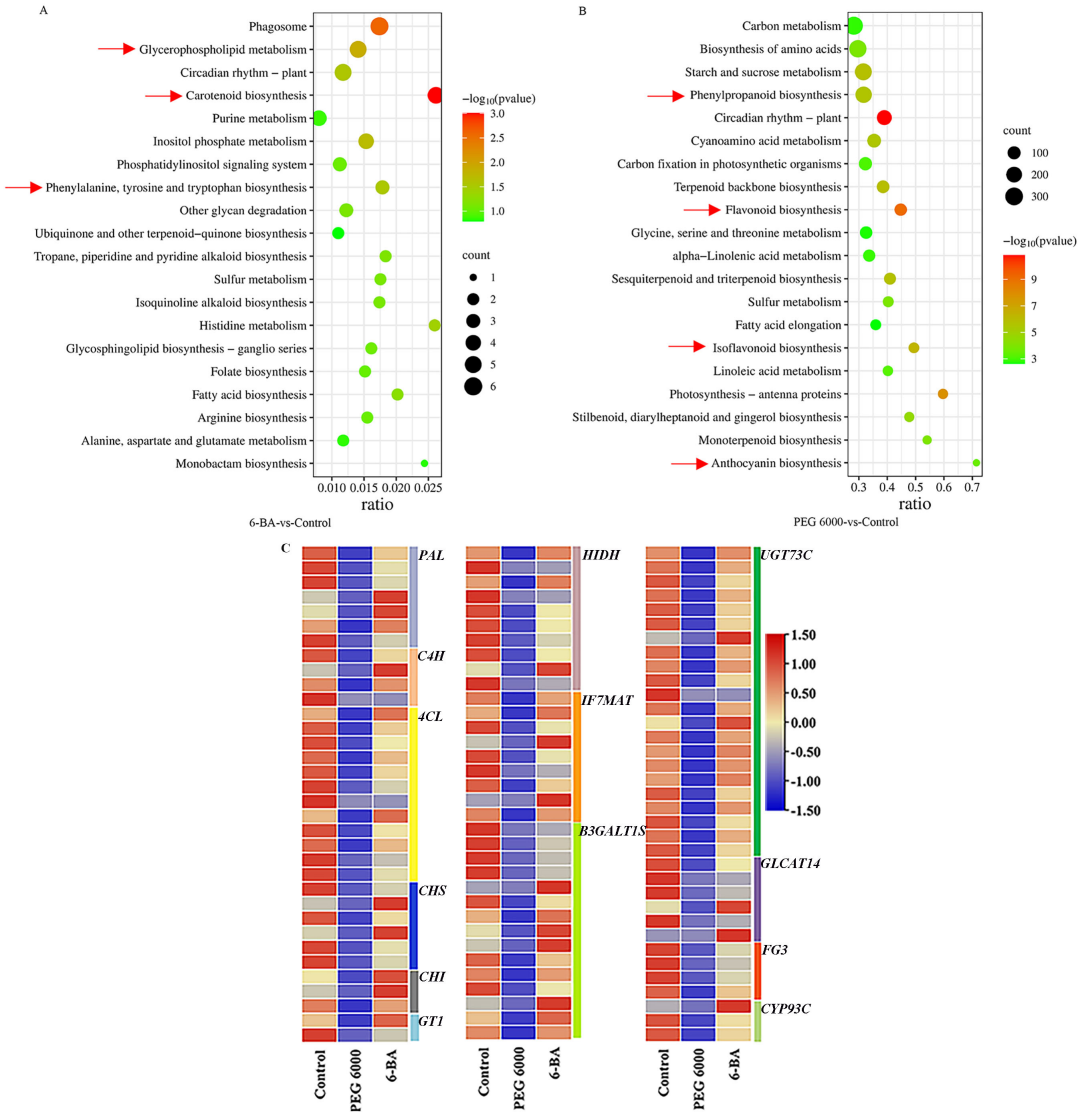
Analysis of differentially expressed genes. **(A)** KEGG functional enrichment of differentially expressed genes between 6-BA and the control. **(B)** KEGG functional enrichment of differentially expressed genes between PEG 6000 and the control. (The unit of the horizontal coordinate in **(A, B)** is Gene ratio, which indicates the ratio of the number of genes involved in the pathway to the total number of genes in the pathway, and the larger the value is, the more genes enriched the pathway is). **(C)** Heatmap of differentially expressed genes involved in biosynthesis (Each column represents a group of samples, each row represents a gene, and the gene FPKM value z-score indicates the expression size of the gene; red indicates that the gene is highly expressed in the sample, and blue indicates that the expression size is small).

Compared with the control group, we found 168 and 12,542 differentially expressed genes in the 100 mg/L 6-BA treatment and 15% PEG 6000 stress groups, respectively. Of these, 73/95 and 4,865/7,677 were upregulated/downregulated in the two groups, respectively. In order to identify genes differentially expressed, we conducted GO and KEGG enrichment analyses. GO enrichment analysis revealed that the differentially expressed genes were primarily involved in metabolic processes and catalytic activities (**Supplementary Table S2**). KEGG enrichment analysis showed that, after 6-BA treatment, the differentially expressed genes were mainly enriched in the phenylalanine and carotenoid biosynthesis pathways. After PEG 6000 stress, several pathways involved in phenylpropane, flavonoid, flavonol and isoflavonoid biosynthesis were enriched among the differentially expressed genes (**Figures 3A, B**).

As shown in **Figure 3C**, 103 relevant differential genes regulating flavonoid biosynthesis were identified. The cleavage enzyme PAL(phenylalanine), located at the beginning of the flavonoid biosynthesis pathway, is involved in the production of metabolites in the early non-branching part of the pathway. The expression of this gene was significantly downregulated after PEG 6000 stress. The 4CL(4-Coumaric acid-coenzyme A ligase) enzyme is responsible for regulating the production of coumaroyl-coenzyme A, which is the foundation for the formation of the mother nuclear structure of flavonoids, and the expression of this gene was significantly downregulated after the PEG 6000 stress. PEG 6000 significantly downregulated the expression of *CHS* (chalcone synthase) and *CHI* (chalcone isomerase). UDP-glycosyltransferase 73C (UGT73C) binds sugars to lipophilic molecules and promotes the synthesis of flavonoid glycosides. PEG 6000 downregulated the expression of 20 genes in this family. There was no significant difference in expression levels of these genes after 6-BA treatment compared to the control group.

**Figure 4:**
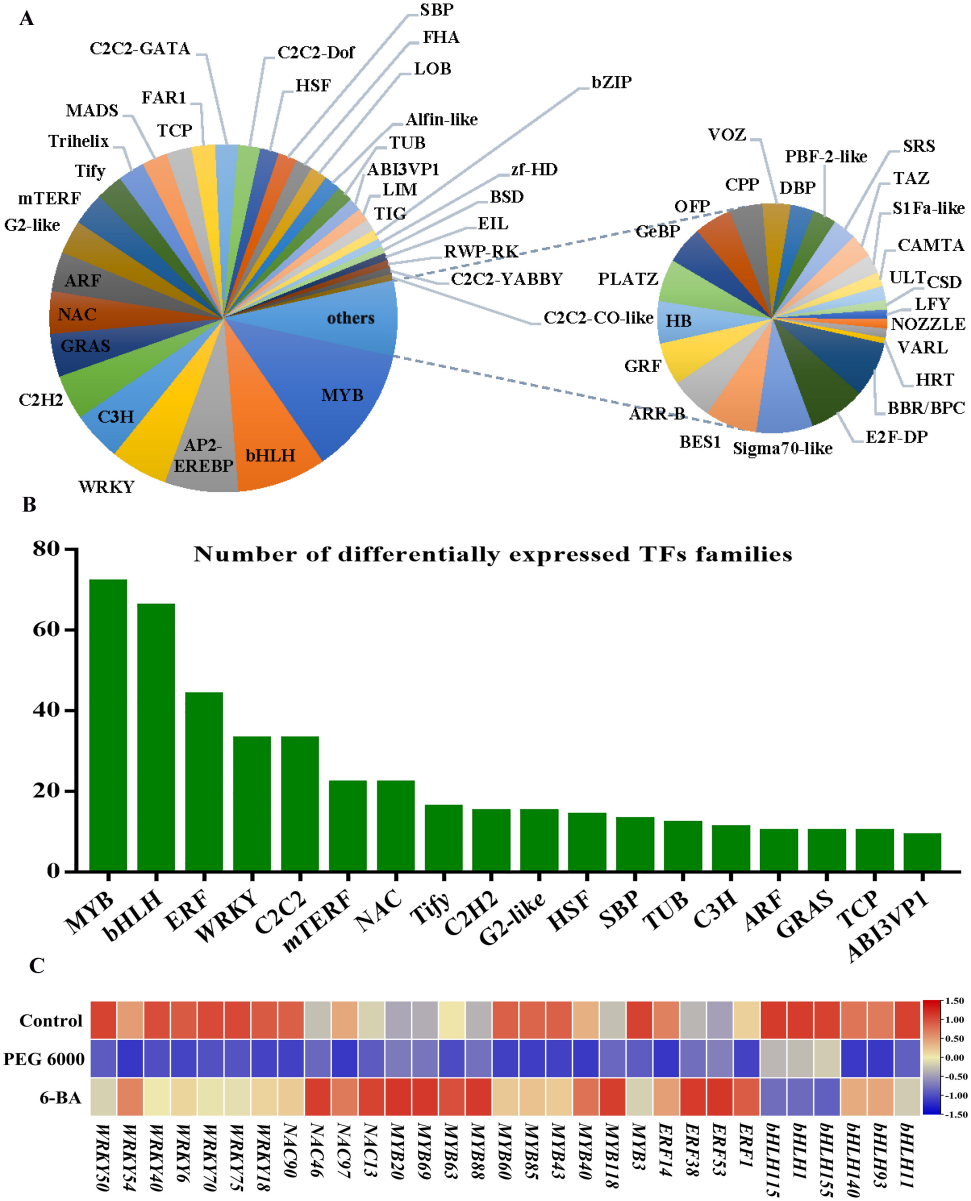
Transcription factor analysis. **(A)** Classification and proportions of transcription factors (TFs). **(B)** Distribution of differentially expressed TF families. **(C)** Heatmap of flavonoid-related differential transcription factors (Each column represents a transcription factor, each row represents a sample, and the amount of expression is indicated by the FPKM value of the transcription factor after the z-score; red indicates that the transcription factor is highly expressed in the sample, and blue indicates that the expression is low).

Transcription factors act as molecular switches that control plant growth and developmental processes under various conditions (Huan et al., 2023). The prediction of genes that encode transcription factors identified 2,178 transcription factors belonging to 58 families (**Figure 4A**). Among them, 522 differentially expressed TFs were identified. Most of the differentially expressed TFs belonged to the MYB, bHLH, and WRKY families (**Figure 4B**). Furthermore, 31 TFs were closely associated with flavonoid metabolite biosynthesis, and their expression levels were significantly reduced after PEG 6000 stress compared with control and 6-BA treatment (**Figure 4C**).

### 2.4 Combined metabolomics and transcriptomics analyses to reveal the mechanism of flavonoid production

**Figure 5:**
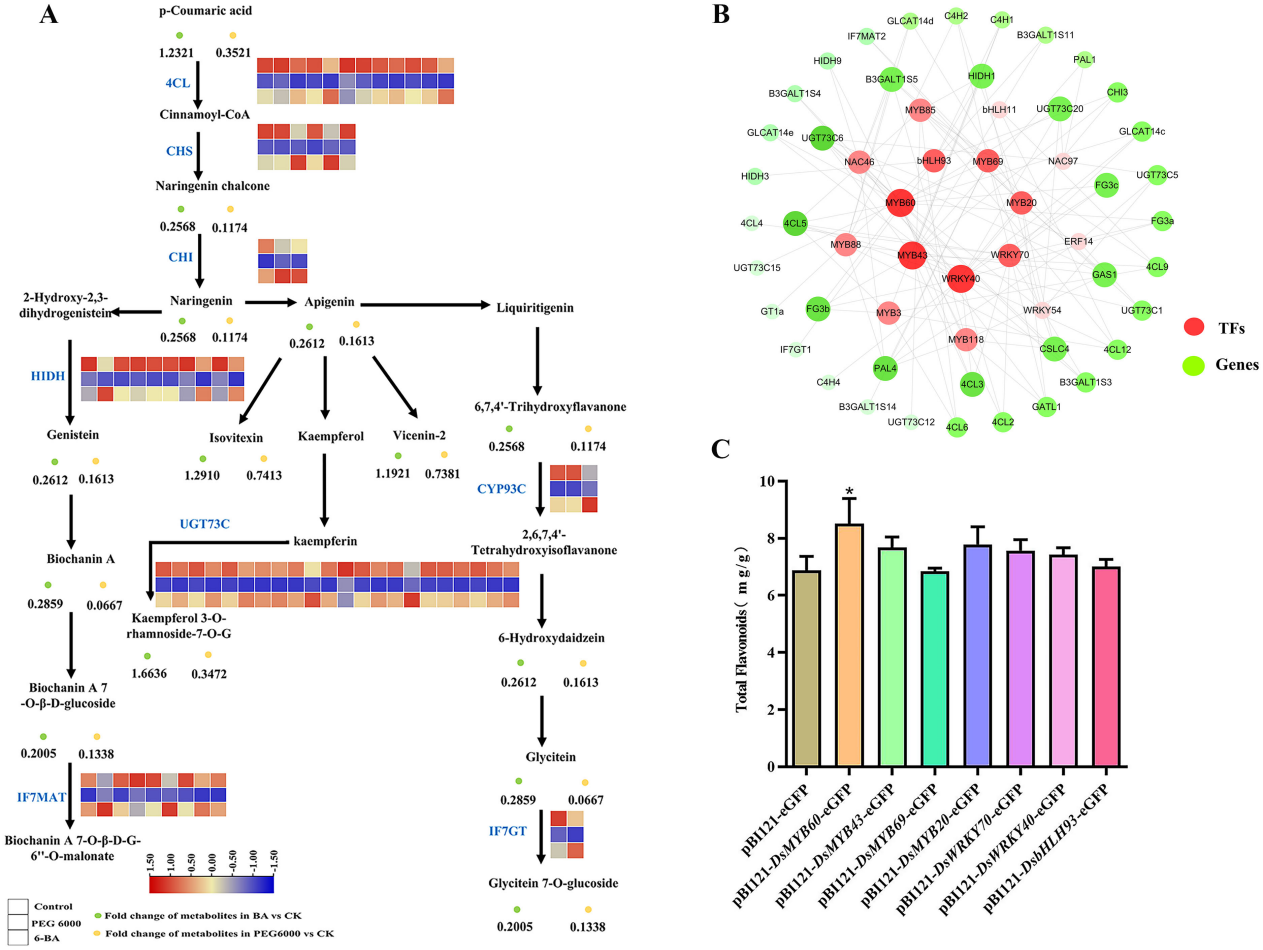
Analysis of the molecular mechanism of flavonoid production and its related transcription factors. **(A)** Combined transcriptome and metabolome analysis of flavonoid compound synthesis pathways. **(B)** Network diagram of the correlations between transcription factors and genes. **(C)** Analysis of total flavonoids in the transient infected leaves of *Nicotiana benthamiana* (*P < 0.05).

Based on metabolite intensity and gene expression data, we focused on the biosynthetic pathways of several compounds, including apigenin, isovitexin, vincristine-2, kaempferol 3-glucoside-7-rhamnoside, and 7,4-dihydroxyflavonoid. We also studied the related enzyme genes regulating these pathways and calculated the correlation between TFs and key structural genes using Pearson’s correlation analysis. Transcriptomic and metabolomic analyses showed no significant difference in the flavonoid metabolic pathway after 6-BA treatment compared to the control group. PEG 6000 coercion induced the differential expression of genes related to the synthesis of flavonoid metabolites, and the expression of genes such as *PAL*, *4CL*, *CHS*, *CHI*, and *UGTs* was downregulated (**Figure 5A**). Based on the correlation coefficients (R > 0.85, positive correlation) between the structural genes and the TFs regulating their expression, a network co-expression view was constructed (using cytoscape 3.8.2) and seven core TFs were identified, as shown in **Figure 5B**.

### 2.5 Functional validation of key transcription factors regulating flavonoid biosynthesis

Overexpression vectors for the seven core TFs were constructed and transformed into Agrobacterium cells. The cloned target genes were identified through agarose gel electrophoresis and sequencing. The results of the evolutionary tree analysis (**Supplementary Figure S3**) of the seven TFs showed a high degree of similarity to the *Arabidopsis thaliana* family of MYB, WRKY, and bHLH TFs. Transient tobacco expression can be used to detect the expression of newly fused genes and to obtain target information for unknown proteins (Mohanan et al., 2023). Therefore, transient tobacco expression was used to functionally validate the seven TFs. As shown in **Figure 5C**, in comparison to the null control, tobacco leaves transfected with the *DsMYB60* gene have a 23.8% increase in the total flavonoid content.

## 3 Discussion

The total flavonoids of *D. styracifolium* (TFDS) were obtained by purification from *D. styracifolium*. TFDS capsules were approved by the NMPA(National Medical Products Administration) for the treatment of urolithiasis in 2022 (Li et al., 2022b). However, the molecular mechanism of flavonoid biosynthesis, which is the main pharmacodynamic component of *D. styracifolium*, remains unclear.

### 3.1 Abiotic stresses lead to an increase in flavonoid content in *D. styracifolium*

In this study, we showed that the TFDS under PEG 6000 stress gradually increased with increasing PEG 6000 concentration (**Figure 1A**). This is similar to the results of Huang et al (Huang et al., 2023), who studied the changes in the flavonoid content of *Sophora alopecuroides* L. under drought stress. The main reason for these results may be that drought stress leads to an increase in the content of ROS (reactive oxygen species), which in turn triggers peroxidation of membrane lipids. Consequently, plants can maintain the stability of the membrane plasmodesmata by accumulating flavonoids. However, the degree of decline in flavonol compounds, such as schaftoside and isovitexin, was proportional to drought intensity (**Figure 1B**); this is similar to the findings of Juan et al (Gutierrez-Gonzalez et al., 2010). In the present study, drought stress increased TFDS (**Figure 1A**), and it has been found that plants such as *Nicotiana tabacum* L. (Cirillo et al., 2021) and *Lycoris radiata* (L’Her.) Herb. (Wang et al., 2023) defend themselves against drought stress by increasing the content of anthocyanins, and because there is competition in the biosynthesis of anthocyanins and flavonols (Cheng et al., 2022), it is speculated that the significant increase in the content of anthocyanins in *D. styracifolium* under drought stress is responsible for the decrease in flavonol compounds, which is the cause of the decrease in flavonols.

In our study, 6-BA treatment promoted the accumulation of total flavonoids and flavonols, which was most significant at low concentrations. In accordance with our findings, exogenous 6-BA treatment increased Rut, IQ, and Ast content in *Morus alba* L. leaves and upregulated *MaF3GT*, *Ma4CL*, *MaPAL*, and *MaCHS*, which are flavonol synthases (Zhang et al., 2021). Therefore, it has been hypothesized that 6-BA treatment induces the expression of relevant genes that regulate flavonoid synthesis in *D. styracifolium*, thereby promoting their accumulation.

In this study, salt stress mainly affected the accumulation of isoorientin and isovitexin glycosides in *D. styracifolium*. This may be because salt stress leads to the enhancement of the oxidative system, which produces more free radicals, and flavonoids accumulate to scavenge excess free radicals. Zhu et al. (Zhu et al., 2021) found that *Sophora alopecuroides* L. defends against salt stress by accumulating lignin and flavonoids, similar to the results of our study.

### 3.2 Integrated transcriptomics and metabolomics analysis reveals the molecular mechanism of flavonoid synthesis in *D. styracifolium*

A recent study showed that the anthocyanin biosynthesis pathway in *Solanum lycopersicum* L. is differentially regulated under different stress conditions (Tapia et al., 2022). In our study, metabolomic analysis showed that differential metabolites were mainly enriched in the flavonoid, flavonol, and isoflavonoid biosynthesis pathway and phenylpropanoid metabolism pathway after PEG 6000 stress (**Figures 2A, B**), and that PEG 6000 stress promoted the accumulation of anthocyanidins and some flavonoids and inhibited the accumulation of flavonols (**Figure 2C**). This is consistent with the findings of this study, which investigated the effects of PEG 6000 on the flavonoid content of *D. styracifolium*. Transcriptome analysis indicated that the differentially expressed genes were enriched in the phenylpropane biosynthesis pathway and the flavonoid, flavonol, and isoflavonoid biosynthesis pathways after PEG 6000 stress (**Figures 3A, B**). Zhang S. K. et al. (2023) conducted research to determine the key genes and secondary metabolites of *Casuarina equisetifolia* Forset. in its response to drought stress, and found that they were similar to those identified in our study. Other studies have shown that drought reduces the expression of biosynthesis-related genes, such as those responsible for lignin and flavonoids in *Polygonatum sibiricum* Delar.ex Redoute (Qian et al., 2021). The results of this study were similar, with the expression of the flavonoid synthesis-related genes *PAL, 4CL, CHS, CHI*, and *UGTs* being downregulated after exposure to PEG 6000 stress (**Figure 3C**). This was consistent with the trend of changes in the contents of their regulated metabolites, which could explain the decreased content of several compounds, such as flavonols and isoflavonoids, in *D. styracifolium* under PEG 6000 stress. Differential metabolites were found to be primarily enriched in metabolic pathways, secondary metabolite synthesis pathways, and the flavonoid, flavonol, and isoflavonoid biosynthesis pathways; in contrast, differential genes were enriched in the phenylalanine and carotenoid biosynthesis pathways after 6-BA treatment. This is inconsistent with previous theories. Comprehensive analysis of the transcriptome and metabolome revealed a strong correlation between gene expression and metabolic profiles in the PEG 6000 stress and control groups. This correlation can help reveal the mechanisms underlying changes in flavonoid metabolism.

### 3.3 Mining of transcription factors and their functional validation

Flavonoid biosynthesis is primarily controlled by structural genes. Transcription factors regulate structural gene expression and are also regulated by other TFs to regulate their synthesis. For instance, it has been demonstrated that *Arabidopsis* flavonols are positively regulated by AtMYB111 under salt stress, which reduces ROS accumulation and helps the plant resist salt stress (Li et al., 2019). In a study of tanshinone metabolism in the traditional Chinese medicine *Salvia miltiorrhiza* Bge. (Li Q. et al., 2022), it was found that SmMYB16 directly binds to the promoters of the tanshinone biosynthesis enzyme genes *SmGGPPS1*, *SmDXS2*, and *SmCPS1* and promotes their expression. In this study, 31 differential TFs regulating the biosynthesis of flavonoid metabolites were identified, belonging to the MYB, WRKY, NAC, bHLH, and ERF transcription factor families (**Figure 4**). Combining the results of metabolomics and transcriptomics, the present study revealed the metabolic pathway of flavonoids in *D. styracifolium* (**Figure 5A**) and found that PEG 6000 stress inhibited the accumulation of metabolites, such as apigenin, isodurin, and vizenin, which was consistent with the trend of changes in the genes related to the synthesis pathway, such as *4CL*, *CHS*, *CHI*, and *HIDH*. Therefore, Pearson’s correlation analysis of the above genes with TFs yielded seven TFs that were significantly correlated (**Figure 5B**): *DsMYB20* (Unigene7277_All), *DsMYB60* (Unigene16997_All), *DsMYB69* (Unigene7213_All), *DsMYB43* (Unigene1052_All), *DsbHLH93* (CL823.Contig7_All), *DsWRKY40* (Unigene602_All), and *DsWRKY70* (CL3866.Contig3_All). Based on the co-expression network diagram, this study suggests that the TFs MYB60, MYB43, and WRKY40 most likely promote the accumulation of flavonoids in *D. styracifolium* by regulating the expression of genes, such as *UGT73C6*, *4CL*, and *HIDH*. Therefore, we constructed overexpression vectors for seven core TFs and transiently infected *Nicotiana benthamiana.* The results showed that DsMYB60 positively affected the synthesis of total flavonoids in *N. benthamiana* leaves (**Figure 5C**). This is the only preliminary functional verification of the TFs.

## 4 Materials and methods

### 4.1 Plant material and growing conditions

The plants were collected from the planting base of *D. styracifolium* (22°46′56′′N, 111°45′55′′E) in the town of Pingtang in the city of Yunfu, Guangdong Province, China. The samples were treated with PEG 6000 (concentrations of 5%, 10%, and 15%) to simulate drought stress, salt stress(concentrations of 5%, 10%, and 15%), and 6-BA(concentrations of 100mg/L, 200mg/L, and 500mg/L) on July 3, 2021, watering 500ml of stress solution once a week, and the coercion will last for 3 months. After treating the leaves with PEG 6000, NaCl, and 6-BA, we collected control leaves, leaves treated with 100 mg/L 6-BA, and leaves treated with 15% PEG 6000.

### 4.2 Determination of total flavonoid and flavonoid glycoside content

*Desmodium styracifolium* leaves were dried in an oven at 80°C until they reached a constant weight. The dried leaves were crushed and passed through a No. 3 sieve. Total flavonoid content was determined using the sodium nitrite-aluminum nitrate method (Petr et al., 2018).

The contents of schaftoside, isovitexin, isoschaftoside, and isoorientin were determined using high-performance liquid chromatography (Guo et al., 2015). Next, 0.2 g of *D. styracifolium* powder was weighed precisely, and 8 mL of an 80% methanol solution was added, extracted by ultrasonication for 30 min, and centrifuged. Then, 1 mL of the supernatant was passed through a 0.22 μm organic filter membrane, and determination was carried out on a Symmetry C18 column (250 mm × 4.6 mm, 5 μ m) with acetonitrile–0.05% formic acid water (15:85) as the mobile phase, a flow rate of 0.6 mL/min, a column temperature of 30°C, and a detection wavelength of 272 nm. The injection volume was 10 µL.

### 4.3 Non-targeted metabolic analysis samples

Six biological replicates per group of biological samples were collected from the control group, the group treated with 6-BA(100 mg/L), and the group subjected to 15% PEG 6000 stress. These samples were then sent to BGI for untargeted metabolic analysis according to the method described by Gao et al (Gao et al., 2020). The sample was separated using an ACQUITY UPLC HSS T3 column (Waters, UK) measuring 100 mm × 2.1 mm in size, with a 1.8-μm particle size. Ultrapure water containing 0.1% formic acid was used as mobile phase A, while mobile phase B consisted of methanol containing 0.1% formic acid. The temperature of the column was set to 50°C, and the flow rate was 0.4 mL/min.

Using high-resolution tandem mass spectrometry (Xevo G2-XS QTOF, Waters, UK) in both positive and negative ion modes, the small molecules eluted from the column were acquired. Centroid data were acquired in MSE mode with a first-stage scanning range of 50–1200 Da and a scanning time of 0.2 s. All parent ions were fragmented at energies ranging from 20 to 40 eV, and all fragment information was collected with a scan time of 0.2 s.

### 4.4 RNA extraction and sequencing

Using the method proposed by Zhao et al (Zhao et al., 2022), nine biological samples collected from the control, 100 mg/L 6-BA-treated, and 15% PEG 6000-stressed (Three biological replicates per group) *D. styracifolium* were sent to the BGI DNBSEQ sequencing platform for transcriptome sequencing. Total RNA was extracted using the RNA *de novo* analysis method provided by BGI Technical Support. An Agilent 2100 was used to determine the concentration of total RNA as well as the 28S/18S ratio and RIN values to ensure quality control. Qualified total RNA was used to construct cDNA libraries, and the specific procedure is shown in the RNA *de novo* analysis method; the mRNA library preparation procedure, including library construction and sequencing, was provided by BGI Technical Support. DNBSEQ sequencing was also performed. The data obtained from sequencing were filtered using the filtering software SOAPnuke (v1.4.0) and the clean reads were denovo assembled using Trinity (v2.0.6) while the assembly quality was assessed by BUSCO. Then, expression levels of genes and transcripts were calculated using RSEM (v1.2.8) software; we annotated the Unigene obtained from transcriptome assembly with seven functional databases (KEGG, GO, NR, NT, SwissProt, Pfam, and KOG), and then used PlantTFDB software for transcription factor prediction.

### 4.5 Phylogenetic tree analysis

By using the Paschalia et al. (Kapli et al., 2020) phylogenetic tree method, a tree of evolutionary relationships was constructed. The Gene IDs of the obtained TFs were used to identify the respective transcription factor family within the Transcriptome Interactive Reporting System and to download the complete amino acid sequences of the proteins they encode. The protein-coding sequences for the transcription factor from the model plant Arabidopsis thaliana were downloaded from the NCBI database. We used the Clustal W program, which is built into MEGA 11.0, to analyze the TFs of *D. styracifolium*. Based on the results, a neighbor-joining phylogenetic tree was constructed.

### 4.6 *Nicotiana benthamiana* instant conversion

Tobacco culture was carried out by sowing seeds purchased from Hua Yue Yang Biotechnology, Beijing. *Nicotiana benthamiana* seeds were planted in sterilized nutrient-rich soil. As described by Buschmann, tobacco transient transformation was performed (Buschmann et al., 2011). The plate of GV3101 strain containing the target gene was removed from the –80°C ultra-low temperature refrigerator and activated through scratching. After the bacteria grew, single colonies were selected and incubated in 2 mL of medium containing kanamycin and rifampicin while shaking. Next, 1 mL of the bacterial solution was inoculated into 35 mL of liquid LB medium, and the resuspension of bacteria was obtained by oscillating the culture. Finally, the bacterium resuspension was drawn into a 1-mL syringe and then injected from the back of the tobacco, allowing the solution to spread through the leaves. The injected tobacco was returned to the incubator for 24 h in the dark and then turned to a low-light culture, and the material was collected after 2 days for subsequent analysis.

## 5 Conclusions

In this paper, we performed different abiotic stresses on *D. styracifolium*, combined metabolomic and transcriptomic analyses, and identified the key regulatory gene *DsMYB60* that regulates the biosynthesis of flavonoid compounds in *D. styracifolium*. Finally, we confirmed that the transcription factor DsMYB60 promotes the production of total flavonoids in *N. benthamiana* leaves through transient expression in *N. benthamiana*. Therefore, we draw a conclusion that *D. styracifolium* regulates the production of flavonoids through DsMYB60 to resist drought stress. These results will be of great significance for further elucidation of the molecular mechanism of flavonoid biosynthesis in *D. styracifolium*, as well as its genetic improvement and comprehensive utilization.

## Data Availability

The data presented in the study are deposited in the NCBI repository, accession number PRJNA1118037.
