# Supplementary material for: Transcriptome-associated metabolomics reveals the molecular mechanism of flavonoid biosynthesis in Desmodium styracifolium (Osbeck.) Merr under abiotic stress
Source: Front Plant Sci. 2024 Aug 19;15:1431148. doi: 10.3389/fpls.2024.1431148 (PMC11366580; doi:10.3389/fpls.2024.1431148)

**Transcriptome-associated metabolomics reveals the molecular mechanism of flavonoid biosynthesis in** ***Desmodium styracifolium* (Osbeck.) Merr under abiotic stress.**

Hongyang Gao^1^, Xi Huang^1^, Pengfei Lin^1,5^, Yuqing Hu^1^, Ziqi Zheng^1^, Quan Yang^1,2,3,4*^

1. School of Chinese Materia Medica, Guangdong Pharmaceutical University, Guangzhou 510006, China

2. Guangdong Provincial Research Center on Good Agricultural Practice & Comprehensive Agricultural Development Engineering Technology of Cantonese Medicinal Materials, Guangzhou 510006, China

3. Comprehensive Experimental Station of Guangzhou, Chinese Material Medica, China Agriculture Research System (CARS-21-16), Guangzhou 510006, China

4. Key Laboratory of State Administration of Traditional Chinese Medicine for Production & Development of Cantonese Medicinal Materials, Guangzhou 510006, China

5.Shenzhen Traditional Chinese Medicine Manufacturing Innovation Ceter Co.,Ltd., Shenzhen, 518110, China

**Corresponding Author**

*E-mail: yangquan@gdpu.edu.cn

Supplementary Material

**1.Supplementary Figures and Tables**

**1.1 Supplementary Tables**

**Supplementary Table S1. Summary of data quality of each sample RNA-Seq**

| **Sample** | **Raw Data** | **Valid Data** | **Q20** | **Q30** | **GC content%** |
| --- | --- | --- | --- | --- | --- |
| **Control_1** | **43.82** | **42.70** | **97.18** | **92.65** | **41.14** |
| **Control_2** | **43.82** | **42.19** | **97.27** | **92.88** | **41.33** |
| **Control_3** | **43.82** | **42.90** | **96.98** | **92.15** | **41.46** |
| **6-BA_1** | **43.82** | **42.64** | **97.09** | **92.44** | **41.18** |
| **6-BA_2** | **43.82** | **42.52** | **97.13** | **92.56** | **41.13** |
| **6-BA_3** | **43.82** | **42.65** | **97.17** | **92.62** | **41.32** |
| **PEG 6000_1** | **43.82** | **42.75** | **97.21** | **92.69** | **40.38** |
| **PEG 6000_2** | **43.82** | **42.56** | **97.41** | **93.20** | **40.93** |
| **PEG 6000_3** | **43.82** | **42.63** | **97.26** | **92.82** | **40.86** |

**Supplementary Table S2.** **GO function annotation of differentially expressed genes**

| **GO classification** | **GO entry** | **Number of annotated genes** | | | |
| --- | --- | --- | --- | --- | --- |
|  |  | **6-BA-vs-Control** | | **PEG 6000-vs-Control** | **PEG 6000-vs-6-BA** |
| **Biological process** | **Biological regulation** | **19** | | **1228** | **949** |
| **Biological process** | **Cellular process** | **66** | | **4408** | **3269** |
| **Biological process** | **Carbon utilization** | - | | **20** | **15** |
| **Biological process** | **Developmental process** | **2** | | **221** | **183** |
| **Biological process** | **Growth** | **1** | | **16** | **13** |
| **Biological process** | **Immune system process** | - | | **28** | **19** |
| **Biological process** | **Interspecies interaction between organisms** | - | | **68** | **55** |
| **Biological process** | **Localization** | **15** | | **704** | **555** |
| **Biological process** | **Metabolic organismal process** | **50** | | **3831** | **2827** |
| **Biological process** | **Muti-organism process** | - | | **176** | **138** |
| **Biological process** | **Multicellular organismal process** | **2** | | **48** | **29** |
| **Biological process** | **Regulation of biological process** | | **17** | **1094** | **837** |
| **GO classification** | **GO entry** | **Number of annotated genes** | | | |
|  |  | **6-BA-vs-Control** | | **PEG 6000-vs-Control** | **PEG 6000-vs-6-BA** |
| **Biological process** | **Negative regulation of biological process** | - | | **105** | **75** |
| **Biological process** | **Positive regulation of biological process** | - | | **153** | **124** |
| **Biological process** | **Reproduction** | **2** | | **120** | **89** |
| **Biological process** | **Reproductive process** | **2** | | **119** | **89** |
| **Biological process** | **Response to stimulus** | **14** | | **980** | **805** |
| **Biological process** | **Rhythmic process** | **1** | | **50** | **46** |
| **Biological process** | **Signaling** | **8** | | **374** | **298** |
| **Cellular component** | **Cellular anatomical entity** | **77** | | **6091** | **4615** |
| **Cellular component** | **Intracellular** | **45** | | **3664** | **2668** |
| **Cellular component** | **Protein-containing complex** | **10** | | **842** | **509** |
| **Molecular function** | **Antioxidant activity** | - | | **75** | **60** |
| **Molecular function** | **Binding** | **69** | | **4749** | **3552** |
| **GO classification** | **GO entry** | **Number of annotated genes** | | | |
|  |  | **6-BA-vs-Control** | | **PEG 6000-vs-Control** | **PEG 6000-vs-6-BA** |
| **Molecular function** | **Catalytic activity** | **73** | | **5123** | **3951** |
| **Molecular function** | **Molecular function regulator** | - | | **153** | **106** |
| **Molecular function** | **Molecular transducer activity** | **3** | | **94** | **75** |
| **Molecular function** | **Nutrient reservoir activity** | - | | **10** | **7** |
| **Molecular function** | **Protein folding chaperone** | - | | **18** | **18** |
| **Molecular function** | **Protein tag** | - | | **9** | **9** |
| **Molecular function** | **Small molecule sensor activity** | - | | **4** | **4** |
| **Molecular function** | **Structural molecule activity** | **2** | | **250** | **77** |
| **Molecular function** | **Transcription regulator activity** | **2** | | **270** | **217** |
| **Molecular function** | **Translation regulator activity** | **2** | | **105** | **55** |
| **Molecular function** | **Transporter activity** | **14** | | **691** | **576** |

**1.2 Supplementary Figures**

**Supplementary Figure S1.** **Unigene length distribution**

**
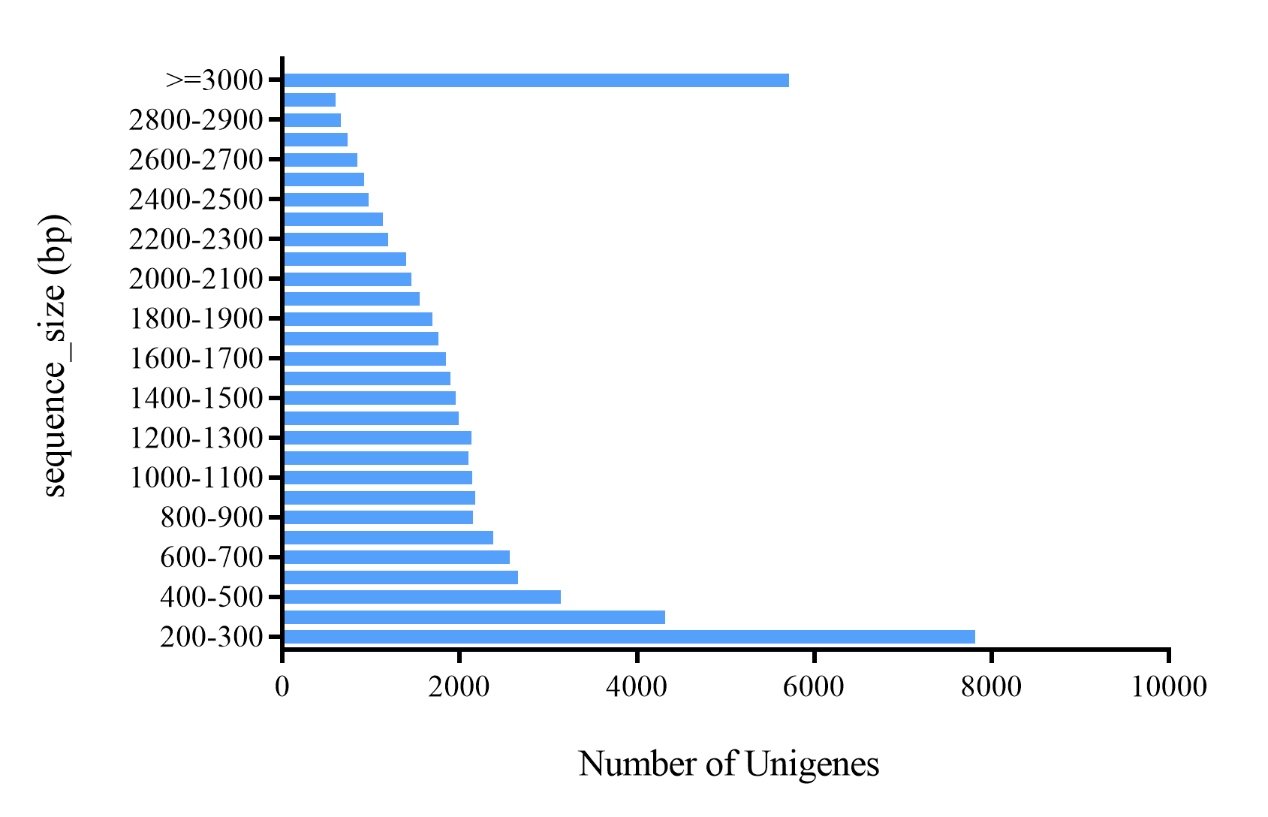
**

**Supplementary Figure S2.** **Real-time quantitative PCR analysis results of 16 target genes compared with RNA-Seq**

**
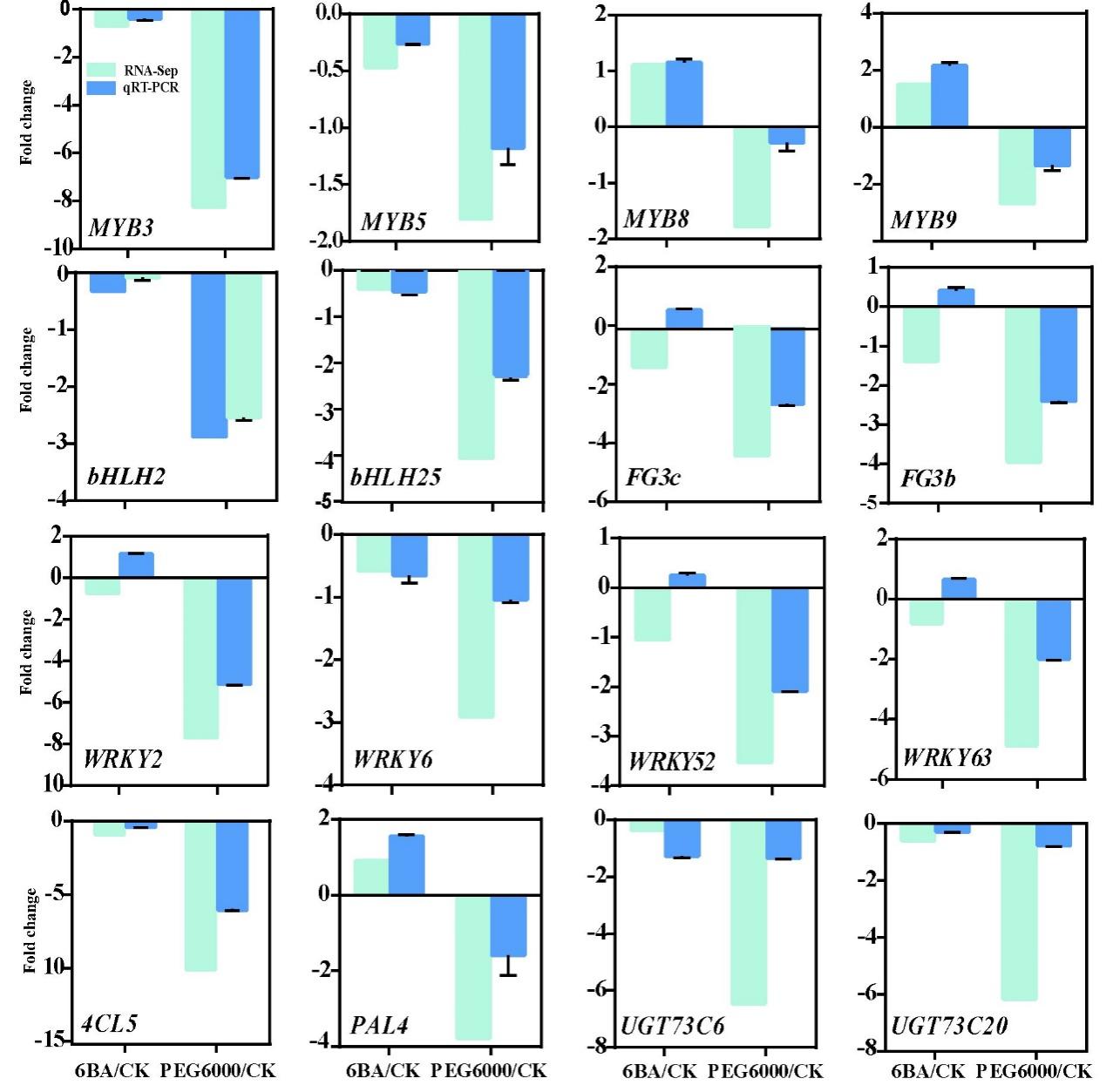
**

**Supplementary Figure S3.** **Analysis of phylogenetic tree of transcription factor alignment with Arabidopsis thaliana**

**A**

**B**
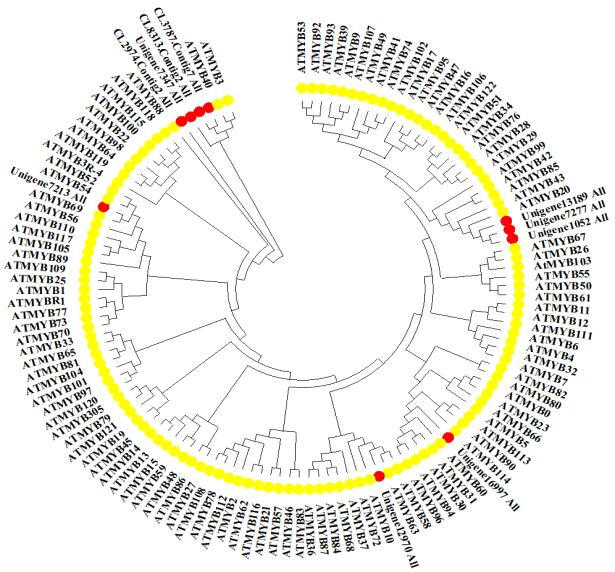


**C**
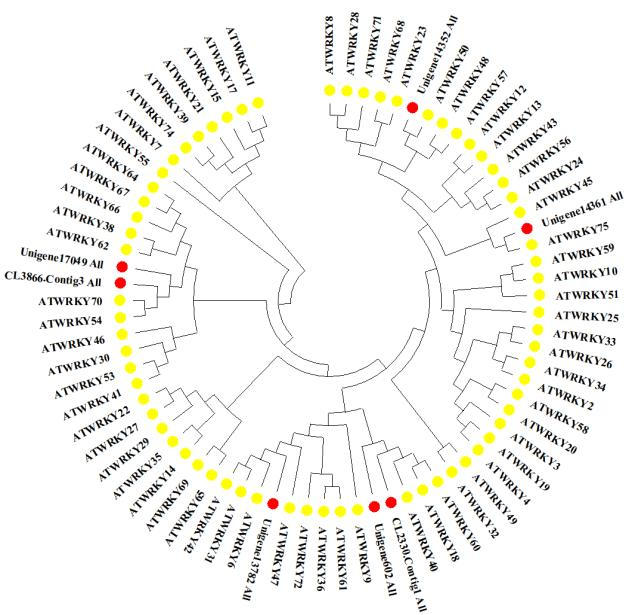


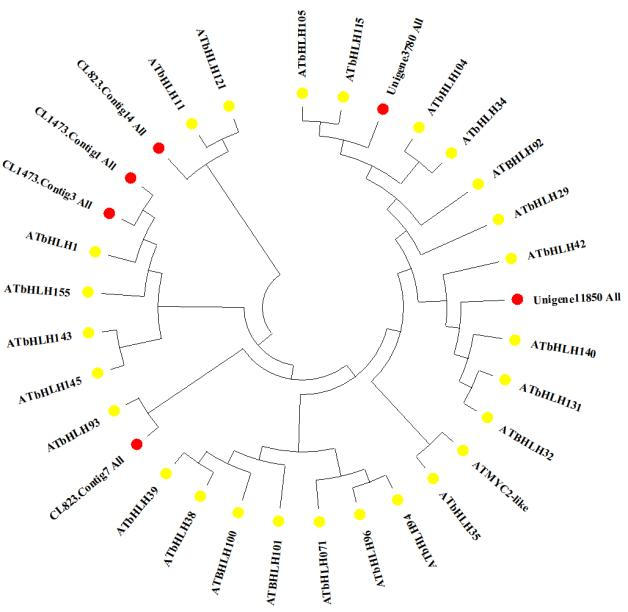

Supplement: Supplementary file 1 [file DataSheet1.docx]
